# Optimization of permeabilization process of yeast cells for catalase activity using response surface methodology

**DOI:** 10.1080/13102818.2014.934986

**Published:** 2015-01-09

**Authors:** Ilona Trawczyńska, Marek Wójcik

**Affiliations:** ^a^ Department of Chemical and Biochemical Engineering, Faculty of Chemical Technology and Engineering, University of Technology and Life Sciences, Bydgoszcz, Poland

**Keywords:** optimization, response surface methodology, yeast, catalase

## Abstract

Biotransformation processes accompanied by whole yeast cells as biocatalyst are a promising area of food industry. Among the chemical sanitizers currently used in food technology, hydrogen peroxide is a very effective microbicidal and bleaching agent. In this paper, permeabilization has been applied to *Saccharomyces cerevisiae* yeast cells aiming at increased intracellular catalase activity for decomposed H_2_O_2_. Ethanol, which is non-toxic, biodegradable and easily available, has been used as permeabilization factor. Response surface methodology (RSM) has been applied in determining the influence of different parameters on permeabilization process. The aim of the study was to find such values of the process parameters that would yield maximum activity of catalase during decomposition of hydrogen peroxide. The optimum operating conditions for permeabilization process obtained by RSM were as follows: 53% (v/v) of ethanol concentration, temperature of 14.8 °C and treatment time of 40 min. After permeabilization, the activity of catalase increased ca. 40 times and its maximum value equalled to 4711 U/g.

## Introduction

Progress in biotechnology enabled wide application of microorganisms in food industry. Biotransformations with the use of intracellular *Saccharomyces cerevisiae* yeast enzymes are well known. Presecki and Vasic-Racki [1] described synthesis of fumaric acid which is widely used in food and beverage products. In addition water molecule to L-malic acid yeast fumarase is employed. Coupling of catalase with oxidases has also been applied for enzymatic production of gluconic acid. Yeast enzymes are not only used directly in the synthesis but also in other stages of food production. Microorganisms can be applied in removing microbicidal and bleaching agent – hydrogen peroxide which is widely used in the food industry.[2] Catalase as an enzyme that belongs to the group of peroxydases is widely applied because of its ability for catalytic decomposition of hydrogen peroxide to water and oxygen.[3–5] *Saccharomyces cerevisiae* yeast is an abundant source of catalase.[4,6–8] Making use of whole yeast cells containing enzyme inside is hindered by small permeability of the cell membrane. Limitation of substrates and products diffusion results in low cell catalytic activity. Enzyme release from the cell core is however connected with expensive purification treatment and partial deactivation. Alternative solution of that problem is to make use of whole yeast cells having increased permeability.

The permeabilization process is one type of cytoplasmatic membrane modification technique. The cell membrane structure is changed under the influence of permeabilization factors in such a way that pores are formed, which would enable free substrate and product diffusion. The cells used in a catalytic process after applying permeabilization can easily be separated from the reaction environment and employed again without fear of greater loss in activity.[6,8] The permeabilization methods of yeast cells have been of interest in many recent publications. Different agents affecting increased catalytic activity of microorganisms have been applied.[9–14] Freeze-thawing method is also used.[15] Among chemical substances the detergent cetyltrimethylammonium bromide (CTAB) was the most frequently employed for permeabilization of *Saccharomyces cerevisiae* [1,4,16–19] and it has been found that CTAB is a substance most effectively enhancing the activity of catalase. This detergent, however, causes fast loss of cell catalytic activity during its storage. Toluene, ethyl ether and alcoholic compounds have often been used in permeabilization process. The first two substances are toxic. Also complete removal of their residuals from the cells after permeabilization is difficult to achieve.[7] Effective treatment of ethanol in studies carried out on different species of *Saccharomyces cerevisiae*, to maintain the activity of two different intracellular enzymes, has been reported.[20,21] The activity of β-galactosidase lightly changed while application of 40% alcohol aqueous solution significantly increased the initial rate of *S*-lactoylglutathione synthesis catalysed by glyoxalase I. Ethanol as a permeabilization reagent has many favourable properties, e.g. it is non-toxic, easily biodegradable and perfectly soluble in water. In the hitherto published papers it has not been considered as a substance enhancing intracellular activity of catalase.

Most works that are devoted to permeabilization address its optimization in relation to the effects of concentration of permeabilization reagent, pH, temperature and treatment time on catalytic activity of the cells. Each of these effects has separately been studied at fixed values of the other parameters. Optimization that is performed in such a way is time-consuming and does not provide a full insight into the effects of significant factors of the process since it does not take into account the effects of mutual interactions. The response surface methodology (RSM) process is applicable in order to determine the interactions between the parameters that influence permeabilization. The method is based on experimental strategy, mathematical methods and statistical reasoning.[22] It makes it possible to assess the effects of parameters leading to an optimal response. The RSM algorithm of action involves simultaneous tests of the variables that significantly affect the process, by using a minimal number of runs, therefore the RSM method is less expensive and less time-consuming as compared to the classical methods.[23] It has successfully been applied for development, improvement and also optimization of biotechnological processes.[24–27] The method has also been used to improve permeabilization processes.[14,28].

The present paper describes a research that aims to optimize the process of permeabilization of *Saccharomyces cerevisiae* yeast cells using ethanol. Because of the presence of intracellular catalase, these whole cell biocatalysts have been successfully used to decompose hydrogen peroxide. We choose to assay intracellular catalase activity using an oxygen electrode. The experiments have been extended to search for an interaction between ethanol concentration, temperature and treatment time of the permeabilization process. The effect of these parameters on the process has then been optimized using the RSM method.

## Materials and methods

Compressed baker's yeast was obtained from Lesaffre Wołczyn, Poland, then was stored and used within 1 week. Anhydrous ethanol 99.8%, hydrogen peroxide 30%, phosphate buffer pH 7 and other chemicals were purchased from POCH S.A. (Polish Chemicals Reagents).

### Permeabilization of yeast cells

Ethanol was used as permeabilization reagent. One gram (wet wt.) of yeast cells was suspended in 20 g of phosphate buffer with organic solvents. Permeabilization effect of different concentrations of alcohol was studied. The contents were mixed and incubated for specified time intervals, under shaking conditions. Afterwards, the cells were analysed for enzyme activity.

### Enzyme assay

Catalytic activity was assayed by measuring the increase in dissolved oxygen resulting from the enzymatic decomposition of hydrogen peroxide, similar to the method described by Delrio et al.[27] Assays were carried out at 20 °C in a jacketed vessel with a total reaction volume of 100 ml H_2_O_2_ solutions with phosphate buffer (pH 7) degassed with N_2_. After adding an appropriate volume of the suspension of permeabilized yeast cells in ethanol, the reactions were started. Small amounts of ethanol did not significantly influence decomposition of hydrogen peroxide. The catalase activity was calculated from the slope of the oxygen concentration versus time profile after baseline subtraction. One unit of enzyme activity is defined as the quantity which degrades 1 μmol of H_2_O_2_ per min under standard conditions.

### Experimental design

Central composite rotatable design (CCRD) proposed by Box is the most accepted and widely used design to study the interaction effect of the medium components. For the determination of the optimal levels of three variables, namely concentration of ethanol, temperature and treatment time on enzyme activity, the response surface approach by using a set of experimental design was performed.[14,29] A total of 20 experiments with eight cube points, six star points (i.e. points having for one factor an axial distance to the centre of ±α, whereas the other two factors are at level 0) and six replicas of the central point (all factors at level 0) were employed to fit the second-order polynomial model. Five different levels for each experiment in coded form are −α, −1, 0, +1, +α, where α = 2^number of variables/4^ = 2^3/4^ = 1.682. The range and levels of experimentally investigated variables are presented in Table 1.

**Table 1.** Values of independent variables at different levels of the central composite rotatable design.

	Real value of variables		
Coded value (level)	Temperature (°C)	Ethanol concentration (%)	Time (min)
−1.682	6.6	24.8	7
−1	10	35	20
0	15	50	40
1	20	65	60
1.682	23.4	75.2	73

The choice of the central plan point, as well as the ranges of measurements, has been made based on the hitherto published papers and preliminary investigations.

### Statistical analysis and optimization

The responses of the CCRD design were fitted with a second-order polynomial equation,(1) 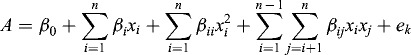
where *A* is the predicted response (dependent variable – enzyme activity) to be modelled. The output variable is characterized by coded, independent input variables (*x*_1_: temperature, *x*_2_: concentration of ethanol, *x*_3_: treatment time). The value of the fitted response at the central point of design, i.e. point (0, 0, 0) is indicated β_0_. The model permitted evaluation of linear β*_i_*, quadratic β*_ii_* and interactive terms β*_ij_* of the independent variables on the dependent variable. Statistical analysis of the mathematical model was performed using ANOVA (analysis of variance). The statistical significance of the second-order model equation was determined by *F*-value. The proportion of variance explained by the obtained model was given by the multiple coefficient of determination, *R* ^2^. Null hypothesis, *H*_0_, was based on an assumption that the angle of slope of the regression line is different from 0. The significance of the regression coefficients was tested by the *t*-test. In order to validate the optimization of medium composition, three tests were carried out using the optimized condition, to confirm the result from the analysis of the response surface. The three-dimensional (3D) response surface curves were then plotted using Statistica 7.1 to explain the interactions effect. Equation was optimized for maximum value to obtain the optimum conditions using Mathcad 15.

## Results and discussion

### Analysing the response

The yeast cells have been permeabilized using ethanol solution under conditions resulting from the assumed experimental strategy. The results of the described randomized experimental design are given in Table 2. The results of the estimated coefficients of Equation (1) together with their statistical assessment at statistically significant >95% confidence level are presented in Table 3. Values of the coefficients in mathematical model for each variable are given together with the result of *t*-Student test at the freedom degree equal to 10. The regression summary indicates that the effect of all the process variables was significant at 5% level (*p* < 0.05) except the linear term of temperature (*x*_1_) and interaction terms of temperature–time (*x*_1_ *x*_3_) and concentration–time (*x*_2_ *x*_3_). High value of correlation coefficient *R* = 0.948 indicates good agreement between the experimental and predicted values. Moreover, the value of the determination coefficient *R* ^2^ = 0.974 points out goodness of regression, which can be used to explain 97.4% of the total variation in the response. The adjusted determination coefficient *R* ^2^(adjusted) differs from the *R* ^2^ only by 0.064 confirming good prediction of the model and demonstrating lack of the so-called effect of the seeming explanation. In order to check consistency of the coefficients of regression, ANOVA analysis has been performed (Table 4).

**Table 2.** Experimental design.

	Temperature *x*_1_		Ethanol concentration *x*_2_		Time *x*_3_		Response *A*
Run	Coded values	Un-coded values (°C)	Coded values	Un-coded values (%)	Coded values	Un-coded values (min)	Enzyme activity (U/g)
1	−1	10	−1	35	−1	20	141.1
2	−1	10	1	65	−1	20	2642.3
3	−1	10	−1	35	1	60	180.5
4	−1	10	1	65	1	60	2886.9
5	1	20	−1	35	−1	20	436.7
6	1	20	1	65	−1	20	1900.7
7	1	20	−1	35	1	60	1801.1
8	1	20	1	65	1	60	1746.5
9	1.682	23.4	0	50	0	40	100
10	−1.682	6.6	0	50	0	40	189.4
11	0	15	1.682	75.2	0	40	941.4
12	0	15	−1.682	24.8	0	40	120.3
13	0	15	0	50	1.682	73	3904.6
14	0	15	0	50	−1.682	6	1631.2
15	0	15	0	50	0	40	4693
16	0	15	0	50	0	40	4707.7
17	0	15	0	50	0	40	4724.6
18	0	15	0	50	0	40	4366.7
19	0	15	0	50	0	40	4523.9
20	0	15	0	50	0	40	4590.1

**Table 3.** Estimated regression coefficients.

Term	Coefficient	SE coefficient	*T* DF = 10	*p*-value
*x*_0_	4583.58	235.77	19.441	0.000
*x*_1_	−19.58	156.42	−0.125	0.903*
*x*_2_	585.58	156.42	3.744	0.004
*x*_3_	389.36	156.42	2.489	0.032
	−1476.39	152.25	−9.697	0.000
	−1323.99	152.25	−8.696	0.000
	−533.27	152.25	−3.503	0.006
*x*_1_*x*_2_	−474.77	204.38	−2.323	0.043
*x*_1_*x*_3_	115.76	204.38	0.566	0.584*
*x*_2_*x*_3_	−164.17	204.38	−0.803	0.440*

Note: *R* = 0.948; *R* ^2^ = 0.974; *R* ^2^(adjusted) = 0.910.

SE coefficient: standard error of coefficient; *T*: test coefficient; DF: degree of freedom.

*Non-significant at 5% level.

**Table 4.** Analysis of variance (ANOVA) for the quadratic model.

Source	SS	DF	MS	*F*-value	*p*-value
Regression	168,083,823	10.00	16,808,382	50.28913	0.0000003
Residual	3,341,684	10.00	334,168		

Note: SS: sum of squares; DF: degree of freedom; MS: mean square.

The results of this analysis validated the mathematical model that describes the permeabilization process. The *F*-test yielding a very low probability level (*F* = 50.289, *p* = 3 × 10^−6^) revealed high statistical relevance of the regression model. The equation of fitted model after neglecting the effect of non-significant terms is as follows:(2) 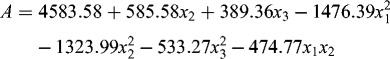



### Optimization of permeabilization process

**Figure 1.**
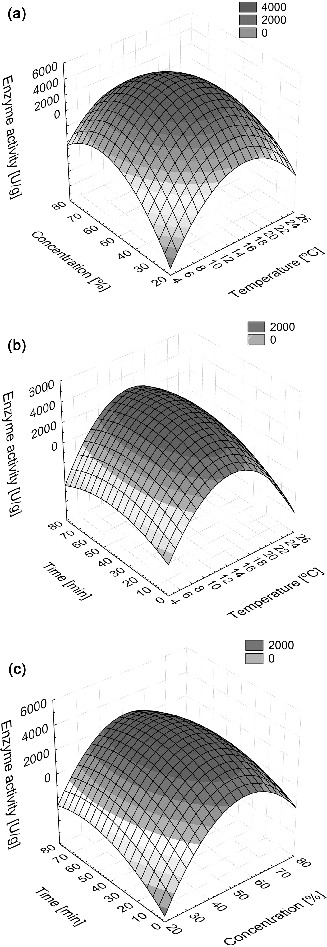
Response surface plots showing the effect of temperature, ethanol concentration and permeabilization time, their combined effects on the enzyme activity (a)–(c); (a) temperature and ethanol concentration, (b) temperature and permeabilization time, (c) ethanol concentration and permeabilization time.

The 3D diagrams of the response surface enable a simple assessment of the system behaviour within the limits of experiment (Figure 1(a)–(c)). The diagrams indicating an effect of two independent variables on a dependent variable assume a constant value of the third variable (at the central point). From a graphical representation of the dependence of catalase activity on temperature, ethanol concentration and time (Figure 1(a)–(c)), one can observe high permeabilization effectiveness within the range of temperature 12–18 °C and ethanol concentration 45%–55%, while below and above these ranges significant decrease of activity can be noticed. The response plane (Figure 1(b) and 1(c)) shows evident maximum within a wide range of time indicating that ethanol concentration and temperature are critical factors. Similar conclusions have been drawn by Panesar from his studies on the ethanol effectiveness in the permeabilization process of the yeast cells *Kluyveromyces marxianus*.[14] Optimization of the process conditions by using numerical techniques enabled determining a maximum value of catalase activity equal to *A*_max_ = 4711 U/g. The corresponding values of temperature, concentration of ethanol and time are 14.8 °C, 53% and 40 min, respectively. Permeabilization of the yeast cells has been conducted and the catalase activity has been measured in the optimal conditions. The obtained value differed from the estimated one by only 5%. The maximum enzyme activity was 40 times higher than the catalytic activity of yeast not treated by the permeabilization process.

The effectiveness of ethanol is slightly lower than that of CTAB.[4] However, in their investigations on the effect of detergent the authors made use of spectrophotometric determination of catalase activity for yeast cells not treated by permeabilization. The method requires using large number of cells in a sample causing disturbance in the measurements of absorbance and at the same time could affect the determination accuracy of the degree of reagent effectiveness.

### Activity of yeast cells during storage

**Figure 2.**
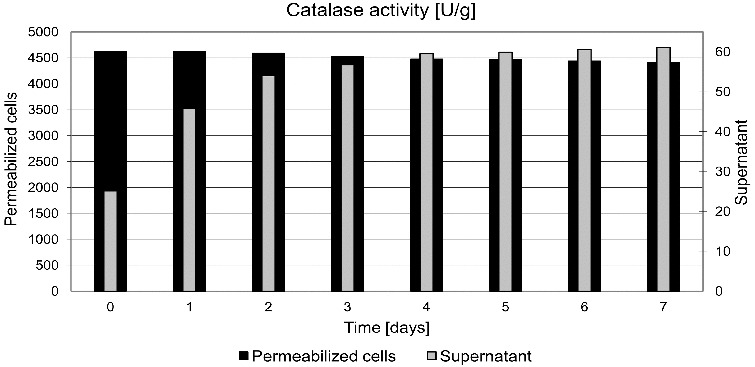
Changed activity of yeast cells during storage.

Yeast cells after permeabilization at the optimal conditions have been tested with respect to maintaining enzymatic activity during storage. The permeabilized cells were dispersed in a phosphate buffer solution with pH 7 and stored at a temperature of 4 °C. Every 24 hours, a predetermined volume of dispersion was collected and separated by centrifugation. Catalase activity was determined in the separated cells and supernatants. A slow decrease of enzyme activity in permeabilized cells and its small increase in supernatant were observed (Figure 2). After storage for 7 days, 85% of the initial activity was maintained. This result is much better than that found for the results of yeast permeabilization using CTAB in which complete loss of catalase activity was noted after 7 days.[4]

## Conclusions

The used permeabilization of yeast cells with ethanol leads to significant increase of the rate of intracellular activity of catalase. Ethanol concentration, temperature and permeabilization time exert influence on that process. The estimated maximum value of the activity of catalase corresponds to a temperature of 14.8 °C, ethanol concentration 53% (v/v) and process duration time of 40 min. The carried out experiments revealed that application of low temperature for permeabilization of the yeast cells with concentrated alcohols is indispensable. Also, it has been proved that the cell storage after permeabilization with ethanol does not lead to significant loss of its catalytic activity. Utilization of ethanol as a permeabilization medium is also beneficial if necessity of solvent removal after process termination is taken into account. The fact that it is a component of a variety of food products enables application of permeabilized cells in the food industry.
